# Autopsy rates and the misclassification of suicide and accident deaths

**DOI:** 10.1007/s10654-024-01142-4

**Published:** 2024-07-24

**Authors:** Jim Schmeckenbecher, Nestor Damian Kapusta, Reinhard Michael Krausz, Christina Alma Emilian

**Affiliations:** 1 Department of Psychoanalysis and Psychotherapy, Medical University, Waehringer Guertel 18-20, 1090 Vienna, Austria; 2 Department of Psychiatry, University of British Columbia, Vancouver, Canada; 3 Department of Psychiatry, University of Oxford, Oxford, UK

**Keywords:** Suicide, Cause of death misclassification, Drug use disorder deaths, Autopsy

## Abstract

Mortality statistics are critical to determine the burden of disease. Certain causes of death are prone to being misclassified on cause of death certificates. This poses a serious risk for public health and safety, as accurate death certificates form the basis for mortality statistics, which in turn are crucial for research, funding allocation and health interventions. This study uses generalised estimating equations and regression modelling to investigate for which cause of death categories suicide and accident deaths are misclassified as. National mortality statistics and autopsy rates from North America and Europe covering the past forty years were analysed to determine the associations between the different causes of death in cross-sectional and longitudinal models. We find that suicides and deaths by accidents are frequently mutually misclassified. We also find that suicides are frequently misclassified as drug use disorder deaths, in contrast to accident deaths, which are not misclassified as drug use disorder deaths. Furthermore, suicides do not seem to be misclassified as undetermined deaths or ill-defined deaths. The frequency of misclassification shows that the quality of death certificates should be improved, and autopsies may be used systematically to control the quality of death certificates.

## Introduction

Suicide and drug use disorder deaths are the leading causes of psychiatric morbidity [1]. These two partially overlapping causes of death are often challenging to distinguish as the determination of suicidal intent in drug use disorder deaths is heavily reliant on suicide notes, which are often not present [2]. Additionally, suicides are specifically prone to being wrongly classified as accidents [3] which results in missing funds for psychiatric care and consequently in absent treatments for both causes of death [1].

Suicide rates differ considerably between countries [4]. Some of this variation may be explained by country-specific differences in suicide risk factors, which may translate into real variations in the prevalence of suicides [5]. However, national suicide statistics rely on official post-mortem certification procedures, which vary across countries. More precisely, after a death occurs, a death certificate is issued indicating the cause of death according to the International Classification of Diseases (ICD). Naturally, countries differ in their national death certification procedures and have varying standards of training, likely affecting the quality and reliability of cause of death statistics [6, 7]. Therefore, heterogeneity in death misclassification can be expected between countries – i.e. some classify more deaths wrongly than others. Literature has suggested that deaths due to suicide are underreported in general and, when compared to other causes of deaths, may be especially subject to misclassification due to the stigma associated with it [8]. Thus, for all these reasons, many studies have questioned the validity of causes of death statistics [9], especially suicide statistics [9, 10], and consequently the validity of all studies relying on official mortality data.

The classic suicide misclassification hypothesis, which emerged in the 1970s, proposes that suicides are `hidden` in other cause-of-death categories [11–13]. Inferentially, the theory assumes that misclassification of suicides should be reflected in a negative association with nonspecific death rates. A previous study [14] has scrutinized this theory and found only weak to no associations between suicides, undetermined-, and ill-defined deaths in cross-sectional and longitudinal analyses. The study then proposed a new suicide misclassification hypothesis introducing autopsies as a covariate to investigate the diagnostic accuracy of death classifications. Doing this, the authors found that autopsy rates were strongly associated with, and predictive of, suicide deaths, drawing doubts on the reliability and validity of death classifications—and consequently of mortality statistics in the presence of low autopsy rates [14].

Based on evidence there are good reasons to assume, that [10]-however, past research failed to confirm the hypothesis that suicides are misclassified as neither deaths of undetermined intent nor as ill-defined deaths. Therefore, it is important to investigate which other death categories may account for these misclassifications. Previous research suggested that one of these categories may be unintentional injury deaths (i.e. accidents) [15]. Indeed, there are scenarios in which suicides and accidents could be confused with each other. For example, a fall from height or a death due to a drug use disorder, need further inquiry to be interpreted as either intentional (suicide) or unintentional (accident) death.

This study has two objectives. First, to examine to what degree suicides and accident deaths are misclassified. We hypothesize that most misclassifications of suicide and accidents occur between each other, given their external similarity but uncertainty of intent. Second, we investigate which other causes of deaths may account for the misclassification of accident and suicides deaths. These causes of death were chosen based on previous literature. More precisely, we investigate the categories undetermined, ill-defined, [12] and drug use disorder deaths [2]. Should these aforementioned causes of death account for the majority of misclassifications, the autopsy rates should no longer be associated with the cause of death in question.

## Methods

This study examines three external causes of death that are differentiated by intention. These three categories are: Intentional injury deaths, unintentional injury deaths, and undetermined/ill-defined deaths. These followed the definition set out in the WHO Mortality Database, with *intentional injury deaths* referring to suicide deaths (ICD-10 codes: X60-X84, Y870) – called “suicides” hereafter. *Unintentional injury deaths* include all kind of accidents (e.g. car accidents) and accidental poisoning – either by medication or alcohol/illegal substances. Hereafter, the former are referred to as “accidents” (V01-X32, X40, X43, X46-X59, Y40-Y86, Y88, Y89, U12.9) and the latter – deaths due to illegal substances and alcohol – as “drug use disorder deaths” (F10-F16, F18-F19, X41-X42, X44, X45). *Deaths of undetermined intent* (Y10-Y34, Y872) are those, where it is not possible to know whether the death of a person was intentional or not. These include cases of death, in which it remains unclear whether an injury death, as described above, was a suicide or an accident. We also include *ill-defined deaths* (R00-R94, R96-R99) – these are deaths, where the death certification procedure did not find a specific cause of death (e.g. unattended deaths, death not otherwise specified). Note that the WHO classification of ICD-10 codes provides a set of mutually exclusive categories [16]. In that WHO classification system drug use disorder deaths should not be confused *drug poisoning.* These are categorized by the WHO into a number of categories, of which the following are relevant for this analysis: “*Suicides*” (i.e., ICD-10, X60–X69: intentional self-poisoning), “*Accidents*” (i.e., ICD-10, X40, X43, X46-X49: accidental poisoning), “*Deaths of undetermined intent*” (i.e., ICD-10, Y10–Y19: poisoning with undetermined intent) and drug use disorder deaths (i.e., ICD-10, X41–X42, X44–X45: accidental drug poisoning not elsewhere classified). Evidently, drug poisoning—as the mechanism of dying – underpins all categories. More precisely, a person can use drug poisoning as a means to commit suicide, poison themselves on accident, the poisoning may be of undetermined intent, or fall under a drug use disorder related death. 

Consequently, as drug poisoning can be the mechanism of death in all the above-mentioned categories, this makes misclassification between these categories more likely. More specifically, these categories may be mutually exclusive in theory but are often hard to distinguish in practice, due to their shared mechanism.

### Data analysis

Data was obtained for the last forty years, i.e. from 1982 until 2022 from the WHO mortality database. Autopsy rates were obtained from the European regional offices’ *European Health for All Database* (https://gateway.euro.who.int/en), the CDC (https://wonder.cdc.gov/) and Statistics Canada (https://www.statcan.gc.ca/en/start). For sensitivity analyses, additional indicators were retrieved from the OECD (https://data-explorer.oecd.org/). To improve robustness, individual years were averaged into periods: For the longitudinal analysis, eight five-year periods, starting with 1982–86, were calculated. For cross-sectional analysis, four ten-year periods, starting with 1982–91, were calculated. 

For the cross-sectional analysis we conducted spearman correlations, bivariate regressions, and multiple regressions. The regressions were used to investigate the associations of each aforementioned variable with suicide and accident deaths. 

Longitudinal data analysis was conducted using generalized estimating equations (GEEs). First, we implemented crude models and then two multiple GEEs were calculated. Similar to the regression models, the GEEs were calculated in order to investigate the associations of each aforementioned variable with both suicide and accidents. 

The formula of both of the multiple regression models and of the multiple GEEs used identity links and a gamma distribution and can be described as following: For the model predicting suicides (per 100.000), the predictors were autopsies (per 100 deaths), drug use disorder deaths (per 100.000), undetermined/ill-defined deaths (per 100.000), and accidents (per 100.000). That is to say: Y(suicides)_i_ = *β*_0_ + *β*_1_ accidents_1i_ + *β*_2_ drug use disorders deaths + *β*_3_ undetermined/ill-defined deaths_3i_ + *β*_4_ autopsy_4i_ + ε_i_). For the model predicting accidents, the predictors were autopsy rates, drug use disorder deaths, undetermined/ill-defined deaths, and suicides (i.e. Y(accidents)_i_ = *β*_0_ + *β*_*1*_ suicides_1i_ + *β*_*2*_ drug use disorder deaths_2i_ + *β*_*3*_ undetermined/ill-defined deaths _3i_ + *β*_*4*_ autopsy_4i_ + ε_i_). Thereby, Y is defined as *μ,* the mean of the outcome Y, moreover *μ* is transformed using the identity link: 1/*μ.* The inclusion of time as an additional predictor to the above-mentioned variables was specific to the longitudinal model.

As accidents and suicides were non-negative and right skewed, gamma distributions were used for both the regression models and GEEs. The correlation structure of the longitudinal model was chosen based on quasi-likelihood under the independence model criterions (QICs) [17].

### Sensitivity analysis

Sensitivity analyses were undertaken to investigate the robustness of results. We investigated (a) data quality, (b) indicator specification and (c) the impact of antidepressant use. Data quality was investigated in two ways: First by limiting countries to only EU member states, and second by only including countries with high data quality according to the WHO data quality classification (https://platform.who.int/mortality/about/data-quality). The impact of indicator specifications was investigated by dividing both undetermined/ill-defined deaths and drug use disorders deaths into their subcategories (ill-defined diseases and ill-defined injuries, and illicit drug use disorder deaths and alcohol use disorder deaths). The effect of antidepressants was investigated by including *defined daily doses*, reported on the national level and provided by the OECD, into the aforementioned models. All analysis was conducted using R version 4.3.2 [18]. This analysis was preregistered on OSF before data retrieval, under: https://osf.io/8pgst.

## Results

**Fig. 1:**
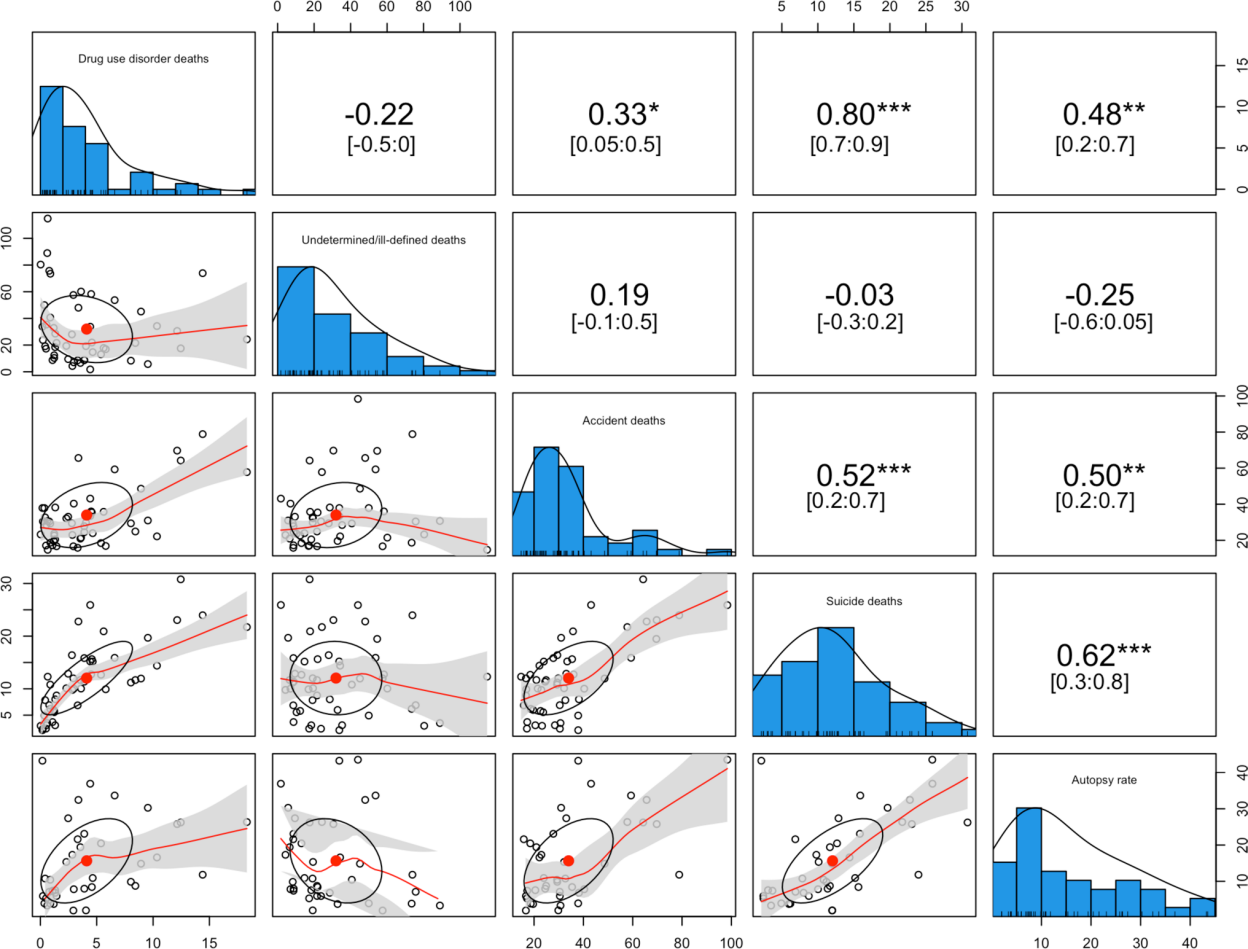
Bivariate associations of variables over all time as Spearman correlations, scatterplots, and histograms. *Note*: Lower triangle: Scatterplot, diagonal histograms; upper triangle spearman: correlations with confidence interval and significance test, ** significant at 0.01, *** significant at < 0.001

Autopsy rates, accidents, and suicides, all declined over the decades. In contrast, undetermined/ill-defined deaths increased strongly between 1982 until 2001 but have since experienced a sharp decline (see Table 1). Correlations between causes of death and autopsy rates were often positive and significant, except for undetermined/ill-defined deaths, which mostly correlated negatively with autopsy rates (see Fig. 1).

**Table 1:** Sample characteristics

Time	Median	25% Quantile	75% Quantile	Range
	Autopsy(as %)			
2022–2012	8.31	5.58	16.32	1.23 – 78.37
2011–2002	9.03	6.14	21.92	1.10 – 47.54
2001–1992	11.46	7.63	26.74	1.68 – 36.58
1991–1982	24.22	11.00	32.94	4.15 – 48.63
	Suicides(per 100,000)			
2022–2012	8.99	5.89	12.17	1.57 – 21.65
2011–2002	10.18	5.87	14.23	1.20 – 30.43
2001–1992	11.72	6.63	16.65	0.42 – 41.60
1991–1982	13.68	7.88	19.50	2.00 – 36.35
	Accidents(per 100,000)			
2022–2012	17.85	13.50	23.41	10.25 – 74.89
2011–2002	23.10	15.79	30.43	13.06 –114.49
2001–1992	30.35	23.27	40.84	12.00 – 120.78
1991–1982	41.15	31.10	51.84	19.28 – 101.36
	Drug use disorder deaths(per 100,000)			
2022–2012	3.84	1.25	7.14	0.24 – 19.95
2011–2002	3.38	1.09	5.65	0.02 – 24.65
2001–1992	3.33	1.49	5.96	0.04 – 15.03
1991–1982	1.57	0.68	2.49	0.00 – 6.39
	Undetermined/ill-defined deaths(per 100,000)			
2022–2012	16.76	9.82	28.30	1.85 – 67.30
2011–2002	22.68	10.58	46.63	1.94 – 155.66
2001–1992	27.91	11.02	56.57	1.84 – 137.66
1991–1982	18.44	11.73	40.16	1.19 – 116.42

Based on all included data

### Regression models

In the bivariate models – averaging results from all four decades – with suicide as the outcome, the strongest association could be observed between suicides and drug use disorder deaths (average of *B* = *1.93 CI*95% [0.96 – 3.08]* p* < 0.001; *R*^*2*^ = 0.45). This was followed by autopsy rates (average of *B* = 0.368* CI*95% [0.20 – 0.56] *p* = 0.03;* R*^*2*^ = 0.33), dropping notably from a substantial *R*^*2*^ = 0.59 in the first decade to a weak *R*^*2*^ = 0.03 [19] in the current decade. Moreover, when using suicide as the outcome, there was a strong association with accidents, explaining an average of *B* = 0.255* CI*95% [0.14 – 0.39] *p* < 0.001;* R*^*2*^ = 0.33, while undetermined/ill-defined deaths were, on average, only associated weakly (*B* = −0.01 *CI*95% [−0.05 – 0.05] *p* = *0.124; R*^*2*^ = 0.06), (see Appendix for full results). Multiple regression models with suicide as the outcome explained on average, over all four decades, an *R*^*2*^ = 0.74 and showed that suicide was strongly associated with drug use disorder deaths and accidents. While the relevance of autopsy rates fluctuated throughout the decades, the impact from deaths of undetermined/ill-defined deaths was negligible over all four decades (see Table 2 for an overview of specific decades).

**Table 2:** Multiple generalised linear models with suicide death as outcome

Predictors	1982–1991		1992–2001		2002–2011		2012–2022	
	Estimates(95%)	*p*	Estimates(95%)	*p*	Estimates(95%)	*p*	Estimates(95%)	*p*
Drug use disorder deaths	0.90(− 0.13–2.20)	0.158	0.73(0.17–1.35)	0.043	0.50(0.12 – 0.97)	0.010	0.57(0.28 – 0.89)	0.002
Accident deaths	0.07(− 0.06–0.21)	0.479	0.20(0.07–0.34	0.022	0.25(0.14 – 0.39)	0.001	0.22(0.09 – 0.37)	0.160
Autopsy rate	0.33(0.15–0.52)	0.016	0.21(0.01–0.41)	0.009	− 0.05(− 0.1 – 0.01)	0.521	− 0.06(− 0.08 – − 0.03)	0.775
Undetermined/ ill-defined deaths	0.00(− 0.04–0.04)	0.954	− 0.01(− 0.06–0.05)	0.774	− 0.05(− 0.06 – − 0.03)	0.079	0.00(− 0.06 – 0.06)	0.983
R^2^ Nagelkerke	0.80		0.81		0.72		0.64	

*p* values are based on robust variance estimations

In the bivariate models – averaging results from all four decades – with accidents as the outcome, the strongest average association with accidents were suicides (average of *B* = 1.27 *CI*95% [0.76 – 1.82] *p* < 0.001; *R*^2^ = 0.42), followed by autopsy rates (average of *B* = 0.85* CI*95% [0.38 – 1.41] *p* = 0.002; *R*^2^ = 0.34) and then drug use disorder deaths (average of *B* = 1.46* CI*95% [−0.048 – 3.27] *p* = 0.11; *R*^2^ = 0.21). As in the models with suicide as the outcome, undetermined/ill-defined deaths were only weakly associated with accidents (average of *B* = 0.11* CI*95% [− 0.06–0.33] *p* = 0.27;* R*^2^ = 0.08), (see Appendix for full results). In multiple regression models with accidents as the outcome, suicides were most strongly associated with accidents – while all other associations were weak over all four decades, together explaining on average an *R*^*2*^ = 0.57 (see Table 3 for an overview of specific decades).

**Table 3:** Multiple generalised linear models with accident deaths as outcome

Predictors	1982–1991		1992–2001		2002–2011		2012–2022	
	Estimates(95%)	*p*	Estimates(95%)	*p*	Estimates(95%)	*p*	Estimates(95%)	*p*
Drug use disorder deaths	− 1.06(− 5.58–4.72)	0.703	0.22(− 1.44–1.99)	0.817	0.05(− 0.73–1.01)	0.924	− 0.11(− 0.80–0.71)	0.758
Suicide	1.92(− 0.20–3.74)	0.099	1.76(0.73–2.79)	< 0.001	1.50(0.62–2.37)	0.046	1.18(0.25–2.12)	0.057
Autopsy rate	− 0.06(− 0.81–0.93)	0.919	− 0.19(− 0.66–0.37)	0.533	0.13(− 0.14–0.63)	0.698	0.10(− 0.05–0.38)	0.627
Undetermined/Ill-defined deaths	0.14(− 0.05–0.39)	0.468	0.15(0.03–0.30)	0.055	0.09(0–0.23)	0.068	0.13(− 0.11–0.39)	0.363
R^2^ Nagelkerke	0.46		0.75		0.62		0.44	

*p* values are based on robust variance estimations

### Generalized estimating equations

**Fig. 2:**
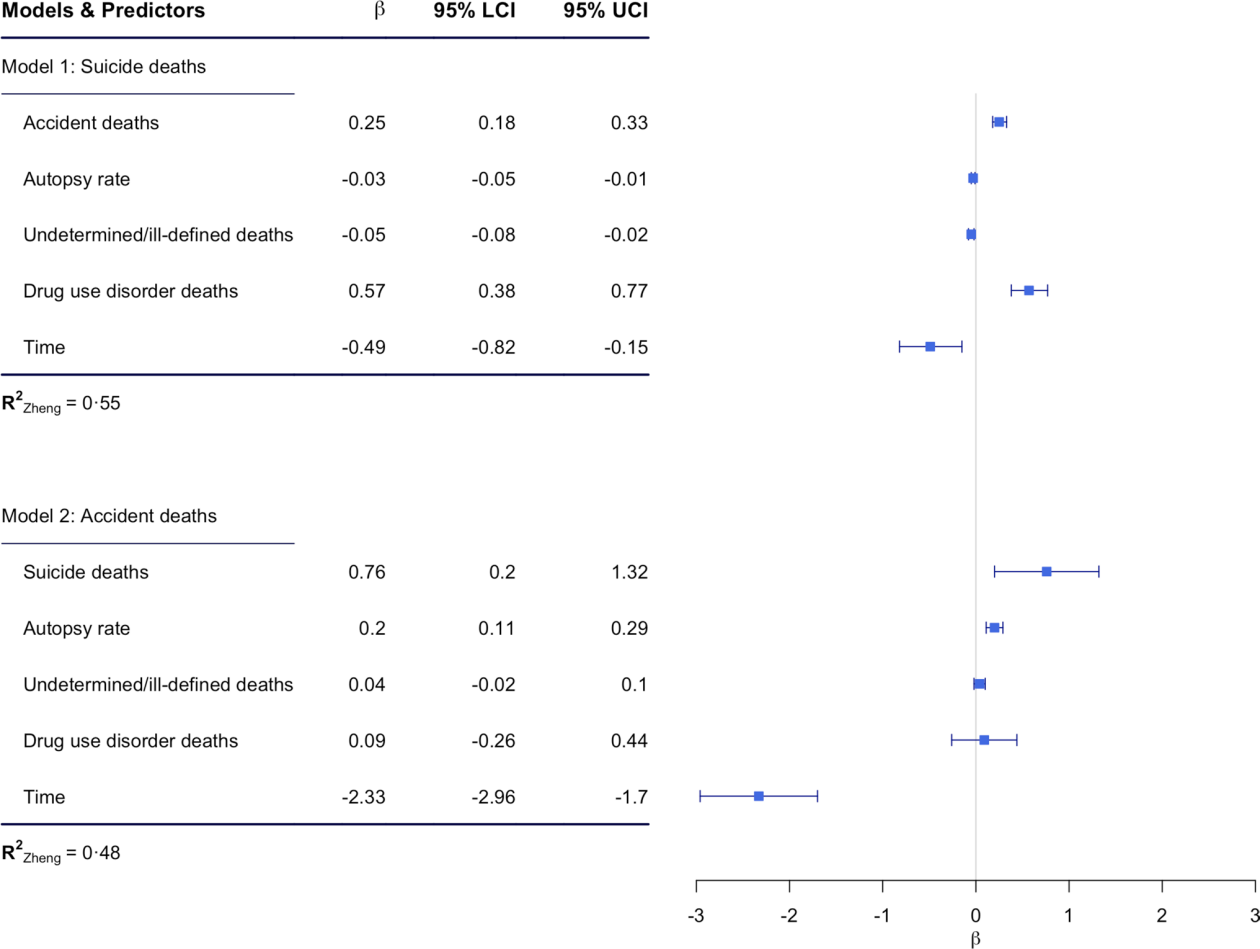
Multiple GEE models. *Note*: Autopsy rate per 100 deaths; causes of death per 100.000 of WHO standardised population

Predicting suicide, the best model fit was achieved with an independence correlation structure (*QIC*: 566). Compared to this, the compound symmetry (i.e., exchangeability) was QIC: *658.8*, and the *QIC* for autoregressive structure was *QIC*: 764. Results of this GEE can be seen in Fig. 2. When comparing the results of the multiple GEE to the bivariate GEEs, the most striking difference was the change of autopsy rates, with a *B* = 0.39 (0.25 – 0.54), *p* < *0*.*001,* and a moderate* R*^2^ = 0.16 in the bivariate model. This means that for every 1% increase in autopsy rates, suicide rates increase by 0.39 per 100,000. In contrast, in the multiple GEE, autopsy rates were significantly associated with a slight decrease of −0.03 per 100,000 suicides. After autopsy rates, the most impacted predictors were undetermined/ill-defined deaths which were not predictive of suicides in the crude GEE (*B* = -0.01CI95% [-0.05 – 0.03] *p* = 0.6; *R*^*2*^ = 0.002)*,* but became significantly associated in the multiple GEE model, predicting that for every unit reduction in undetermined/ill-defined deaths, an increase of 0.05 per 100,000 suicide deaths could be observed. Compared to this, the other bivariate GEE only reported weak changes for drug use disorder deaths [*B* = 1.11 CI95% (0.73 – 1.49) *p* < 0.001 *R*^2^ = 0.12], leading to an increase of 1.11 per 100,000 suicides. Notably, accidents reported the same mean effect size in the bivariate GEE (*B* = 0.25 CI 95% (0.20 – 0.30), *p* < 0.001; *R*^2^ = 0.48) as in the multiple GEE model.

Predicting accident deaths, the multiple GEE achieved the best model fit with an autoregressive structure (*QIC:* 477.13**)**. Compared to this, the compound symmetry (i.e., exchangeability) was QIC: 485.24, and the independent structure was *QIC*: 505.86. Results of this model can be seen in Fig. 2.

Comparing the results of the multiple GEE to the results of the bivariate models, the strongest change was again observed for autopsy rates. In the bivariate GEE, autopsy rates had a strong impact on accident deaths at *B* = 0.72 CI95% (0.42 – 1.03, *p* < 0.001; with a moderate *R*^*2*^ = 0.20) and a 1% change in autopsy rates was associated with an increase of 0.72 per 100,000 in accident deaths. The remaining predictors only changed insignificantly, as the bivariate models, drug use disorder deaths (B = 0.18 [0.02 – 0.33] *p* = 0.69; R^2^ = 0.02), undetermined/ill-defined deaths (B = 0.08 [0.07 – 0.09] *p* = 0.12; *R*^*2*^ = 0.03) and suicide deaths (B = 1.41 CI95% [0.82 – 2.00] *p* < 0.001; R^2^ = 0.43) reported comparable effects to those of the multiple GEE (see Fig. 2). Therefore, in the bivariate GEE predicting accidents, only suicides were able to explain a substantial degree of variance with an R^2^ = 0.43. We implemented a set of sensitivity analysis which affected the above presented results only marginally (see Appendix for full results).

## Discussion

Confirming prior results [14, 20, 21] we fail to find evidence that suicides are mainly misclassified as either undetermined or ill-defined causes of death. Expanding previous investigations to include further causes of death, we find that suicides are misclassified as accident and drug use disorder deaths. Indeed, when accounting for accident deaths, drug use disorder deaths and undetermined/ill-defined deaths, the association between autopsy rates and suicide weakens considerably, implying that accidents and drug use disorder deaths are the two main causes of death which suicides are misclassified as. Furthermore, we find evidence that the direction of the misclassification of accidents and suicides goes both ways, suggesting that accidents are misclassified as suicides and vice versa. However, drug use disorder deaths were only associated with suicides but not with accidents, i.e. suicides are misclassified as drug use disorder deaths, while accident deaths are not misclassified as drug use disorder deaths. Indeed, autopsies were still associated with accidents deaths after accounting for all other causes of death, suggesting that—while we were able to identify the main causes of misclassification for suicides-, there remains unidentified important causes of misclassification for accidents. Altogether, our study supports the idea that suicides and drug use disorder deaths are uniquely prone to conflation with suicides in death statistics.

### Causes for misclassification

Various reasons exist why these causes of death may be confused with each other. Both suicide and accident deaths are regarded as *injury deaths* by the WHO and the only difference between accidents and suicides is whether the deceased had the intent to die or not. In some cases, intent is apparent: For example, when a suicide note is present [2]. In these cases, death classification is more straightforward. However, in other cases, intent may not be as overt. For example, suicides may be misclassified as accidents when a person intentionally jumps from a roof or in front of a car – rather than falling accidentally [6]. 13/07/2024 14:43:00 PMIn such cases, intent can be hard to investigate, in particular when no suicide note has been both left and found [2]. Contrarily, accidents may be classified as suicides as well, for example a person suffering from depression may accidentally shoot themself while handling a firearm, which may be wrongly classified as a suicide due to their history of mental health problems [22].

It is unsurprising that more suicides are being misclassified as accidents than vice versa because the base assumption of an injury death is usually that an accident has occurred [3]. In case no contradicting evidence is present – e.g. in form of suicide notes/announcements [10]—suicides will thus routinely, and therefore at times wrongly, be classified as accidents. This may be aggravated by an overworked medico-legal system, [10] which counteracts deeper case investigations and forces technical and financial shortcuts due to generally limited resources: For one, such investigations are resource intense – e.g. the family of the deceased needs to be interviewed and the medical history has to be reconstructed in a psychological autopsy to evaluate whether it is sensible to assume the individual acted with suicidal intent. Furthermore, suicides are stigmatized, and officials may at times be biased to classify a death as an accident rather than a suicide, given that the former may be a preferable cause of death for the family and others [8]. In short, suicide as a cause of death has a higher burden of proof when compared to other causes of death [3, 10, 23], which, in an overworked system, may lead to an unconscious but preferred misclassification into other cause of death categories which put less strain on the examiners.

### Implications

The accurate assessment of causes of death, or lack thereof, has significant implications. Cause of death statistics – which are based on death certificates—have an important role in the calculation of the burden of diseases and mortality (e.g. disability-adjusted life years [DALYs]), which informs the allocation of resources in the healthcare system—for example towards capacity building for prevention and treatment of mental health disorders and suicide. Thus, accurate death investigations have a decisive indirect effect on valid cause of death statistics and associated health care planning [24].

Autopsy rates have been decreasing in Europe and North America. Our study implies that both suicides and accidents may frequently be misclassified, which has wide ranging consequences. More precisely, when the correct classification of death is reliant on autopsy rates, the comparability of death statistics between countries is limited – i.e. countries with lower autopsy rates tend towards wrongly classifying more suicide and accident deaths than countries with high autopsy rates [25, 26]. Many studies utilize national death statistics and, if these are biased, research using measures derived from this data, such as DALYs, would have limited reliability and validity [9]. If this holds true, the current scientific literature may be less reliable than believed—as may be all public health interventions informed by this data.

Misclassifying causes of death threatens public health and safety and wastes limited public health resources by misallocating funds. Our study emphasises the need to improve cause of death classifications by improving the funding of the medico-legal system and adapting new standards and methods of classifying deaths [27], such as new approaches to categorisations [3], systems [28, 29], or simply better funding for more autopsies to ensure valid classifications of death. Currently, countries do not do this to a satisfactory extent as reflected in the poor quality of death statistics. Furthermore, countries may want to consider using autopsies in unambiguous death cases on a random test basis as a measure for quality control of death certification procedures. This could be used to monitor the accuracy of national death statistics and to inspect the ability of those certifying deaths, thus identifying professions, groups or individuals who require further training [25, 26]. Akin to the WHO data quality statistics [30], these investigations can be openly made available to researchers. More precisely, as long as cause of death misclassifications remain a challenge, we strongly urge that autopsy rates and other methods should be developed and established as a standard measure. Thus, like gender, age, or unemployment, autopsy rates—or other measures of cause of death misclassification—should be broadly included as confounders in the analyses of ecological data. There are limitations to this study. More precisely, as the analyses is based on ecological data, interferences should be made with caution. Moreover medico-legal systems vary between different countries, which makes drawing universal conclusions difficult. Therefore, research should be conducted in individual countries taking differences into account to provide information better tailored to the need of the respective countries and regions.

## Conclusion

Our study suggests that suicides may be misclassified as accidents and drug use disorder deaths, and that accidents may be misclassified as suicides. Consequentially, our results imply that death certificates regularly include errors, thereby biasing national death statistics and thus all measures and studies based on these statistics. Therefore, autopsies should be conducted more frequently in equivocal cases—and on a test basis in unequivocal cases—to improve death certifications. Until these changes have been implemented, the present bias can be minimized by including autopsy rates as a confounder in studies based on death statistics.

## Appendix:

See Tables 4, 5, 6, 7, 8, 9, 10, 11, 12, 13, 14, 15, 16, 17, 18 and 19.

**Table 4:** GLM: Splitting illicit drugs and accidents, with suicide as outcome

Predictors	Suicide: 2022–2012				Suicide: 2011–2002				Suicide: 2001–1992			
	*Estimates*	*p*	*Estimates*	*p*	*Estimates*	*p*	*Estimates*	*p*	*Estimates*	*p*	*Estimates*	*p*
Illicit drug use disorder deaths mean	0.70(0.23 – 1.26)	**0.002**			0.61 (-0.13 – 1.52)	0.107			1.20 (0.08 – 2.66)	**0.050**		
Accident deaths mean	0.33(0.21 – 0.48)	** < 0·001**	0.10 (−0.07 – 0.28)	0.245	0.32(0.21 – 0.46)	** < 0·001**	0.21(0.10 – 0.35)	**0.001**	0.27(0.16 – 0.40)	** < 0·001**	0.16(0.02 – 0.30)	**0.026**
Autopsy rate mean	−0.07(−0.10 – −0.04)	** < 0·001**	−0.06(−0.08 – −0.03)	** < 0·001**	−0.06(−0.11 – 0.01)	**0.025**	−0.06(−0.10 – 0.00)	**0.012**	0.25(0.05 – 0.45)	**0.008**	0.22(0.02 – 0.42)	**0.017**
Ill defined deaths mean	−0.00(−0.06 – 0.06)	0.952	−0.02(−0.08 – 0.05)	0.663	−0.05(−0.07 – −0.03)	** < 0·001**	−0.05(−0.06 – −0.03)	** < 0·001**	−0.01(−0.06 – 0.05)	0.667	−0.02(−0.06 – 0.04)	0.456
Alcohol deaths mean			1.22(0.54 – 1.92)	** < 0·001**			0.93(0.28 – 1.74)	**0.001**			1.04(0.19 – 1.94)	**0.014**
R^2^ Nagelkerke	0.573	0.614	0.686	0.735	0.792	0.799						
AIC	170.416		167.156		189.423		183.887		173.476		176.735	

Bold denotes significance at p = 0.05

**Table 5:** GLM: Splitting illicit drugs and accidents, with accidents as outcome

Predictors	Accidents: 2022–2012				Accidents: 2011–2002				Accidents:2001–1992			
	Estimates	*p*	Estimates	*p*	Estimates	*p*	Estimates	*p*	Estimates	*p*	Estimates	*p*
Alcohol deaths mean	4.34(1.33 – 17.84)	**0.030**			2.06(0.59 – 10.26)	0.324			5.90(0.84 – 46.75)	0.104		
Suicide deaths mean	1.41(0.65 – 3.08)	0.415	3.67(1.89 – 7.25)	** < 0·001**	3.47(1.48 – 8.17)	**0.007**	5.15(2.33 – 10.48)	** < 0·001**	3.52(1.39 – 9.28)	**0.009**	7.20(3.68 – 13.97)	** < 0·001**
Autopsy rate mean	1.09(0.96 – 1.36)	0.268	1.09(0.96 – 1.40)	0.264	1.10(0.86 – 1.75)	0.482	1.10(0.86 – 1.78)	0.462	0.84(0.55 – 1.37)	0.453	0.81(0.54 – 1.31)	0.345
Ill defined deaths mean	1.06(0.90 – 1.30)	0.520	1.06(0.84 – 1.39)	0.586	1.09(1.00 – 1.24)	0.117	1.08(0.99 – 1.23)	0.157	1.15(1.02 – 1.32)	**0.047**	1.14(1.02 – 1.31)	**0.043**
Illicit drug deaths mean			0.58(0.34 – 1.12)	0.137			0.47(0.15 – 2.00)	0.299			0.09(0.02 – 0.68)	**0.019**
R^2^ Nagelkerke	0.536	0.489	0.638	0.638	0.765	0.793						
AIC	220.555		223.647		248.558		248.525		233.777		223.552	

Bold denotes significance at p = 0.05

**Table 6:** GLM Splitting ill-definied and undetermined deaths; suicide as outcome

*Predictors*	**Suicide: 2022–2012**				**Suicide: 2011–2002**				**Suicide: 2001–1992**			
	*Estimates*	*p*	*Estimates*	*p*	*Estimates*	*p*	*Estimates*	*p*	*Estimates*	*p*	*Estimates*	*p*
Drug use disorder deaths mean	1.76(1.32 – 2.44)	** < 0·001**	1.75(1.32 – 2.40)	** < 0·001**	1.78(1.18 – 2.96)	**0.003**	1.63(1.09 – 2.60)	**0.006**	2.13(1.22 – 3.96)	**0.007**	1.62(0.99 – 2.80)	0.063
accident deaths mean	1.25(1.09 – 1.44)	**0.002**	1.25(1.10 – 1.45)	**0.001**	1.29(1.15 – 1.48)	** < 0·001**	1.26(1.14 – 1.42)	** < 0·001**	1.21(1.06 – 1.39)	**0.003**	1.33(1.17 – 1.52)	** < 0·001**
autopsy rate mean	0.94(0.92 – 0.97)	** < 0·001**	0.96(0.90 – 1.02)	0.204	0.94(0.89 – 1.02)	**0.027**	1.04(1.00 – 1.12)	0.064	1.25(1.02 – 1.53)	**0.017**	1.30(1.11 – 1.54)	**0.001**
Ill-defined diseases mean	1.00(0.94 – 1.07)	0.929			0.96(0.94 – 0.99)	** < 0·001**			1.00(0.95 – 1.06)	0.907		
Ill-defined injuries mean			0.91(0.62 – 1.30)	0.637			0.78(0.71 – 0.87)	** < 0·001**			0.57(0.39 – 0.84)	**0.003**
R^2^ Nagelkerke	0.643	0.646	0.672	0.722	0.806	0.852						
AIC	164.671		164.375		190.861		185.447		171.410		163.456	

Bold denotes significance at p = 0.05

**Table 7:** GLM Splitting ill-definied and undetermined deaths; accidents as outcome

*Predictors*	Accidents: 2022–2012				Accidents: 2011–2002				Accidents:2001–1992			
	*Estimates*	*p*	*Estimates*	*p*	*Estimates*	*p*	*Estimates*	*p*	*Estimates*	*p*	*Estimates*	*p*
Drug use disorder deaths mean	0.87(0.46 – 1.86)	0.698	0.90(0.43 – 2.14)	0.788	1.11(0.51 – 2.91)	0.822	1.07(0.48 – 2.87)	0.890	1.23(0.23 – 7.19)	0.807	1.12(0.30 – 5.11)	0.873
Suicide deaths mean	3.13(1.30 – 7.68)	**0.014**	3.25(1.22 – 8.60)	**0.019**	3.88(1.67 – 8.81)	**0.001**	4.05(1.59 – 9.78)	**0.002**	5.88(2.11 – 16.60)	** < 0·001**	6.89(2.72 – 17.26)	** < 0·001**
Autopsy rate mean	1.12(0.98 – 1.48)	0.177	1.02(0.79 – 1.41)	0.884	1.18(0.90 – 1.95)	0.249	0.94(0.61 – 1.66)	0.726	0.83(0.51 – 1.48)	0.475	0.67(0.44 – 1.07)	0.063
Ill-defined diseases mean	1.17(0.96 – 1.49)	0.172			1.11(1.00 – 1.31)	0.096			1.16(1.02 – 1.36)	0.058		
Ill-defined injuries mean			1.83(0.63 – 6.57)	0.350			1.60(0.86 – 4.18)	0.174			8.45(2.74 – 32.43)	** < 0·001**
R^2^ Nagelkerke	0.460		0.439		0.624		0.594		0.738		0.815	
AIC	225.405		226.651		249.810		252.314		230.449		220.298	

Bold denotes significance at p = 0.05

**Table 8:** GLM anti-depressants and autopsy rates on suicides and accidents

*Predictors*	Suicide: 2022–2012		Suicide: 2011–2002		Suicide: 2001–1992		Accidents: 2022–2012		Accidents:2011–2002		Accidents: 2001–1992	
	*Estimates*	*p*	*Estimates*	*p*	*Estimates*	*p*	*Estimates*	*p*	*Estimates*	*p*	*Estimates*	*p*
Autopsy rate mean	0.24(0.10 – 0.41)	**0.002**	0.26(0.14 – 0.38)	** < 0·001**	0.48(0.22 – 0.76)	** < 0·001**	0.25(0.05 – 0.47)	**0.010**	0.37(0.09 – 0.67)	**0.008**	0.38(−0.41 – 1.13)	0.317
Anti depressants mean	0.02(−0.01 – 0.07)	0.232	0.00(−0.05 – 0.06)	0.912	−0.06(−0.23 – 0.09)	0.482	−0.05(−0.09 – −0.01)	**0.017**	−0.13(−0.23 – −0.02)	**0.019**	−0.56(−1.02 – −0.12)	**0.015**
Observations	17		16		12		17		16		12	
R^2^ Nagelkerke	0.399		0.585		0.686		0.529		0.628		0.633	
AIC	84.583		78.949		73.726		88.174		100.813		95.551	

Bold denotes significance at p = 0.05

**Table 9:** Model using data from EU members only

*Predictors*	Suicide: 2022–2012		Suicide: 2011–2002		Suicide: 2001–1992		Accidents:2022–2012		Accidents:2011–2002		Accidents: 2001–1992	
	*Estimates*	*p*	*Estimates*	*p*	*Estimates*	*p*	*Estimates*	*p*	*Estimates*	*p*	*Estimates*	*p*
Drug use disorder deaths mean	0.48(0.18 – 0.80)	**0.001**	0.32(−0.02 – 0.74)	0.062	0.80(0.20 – 1.46)	**0.007**	−0.58(−1.33 – 0.23)	0.130	−0.08(−0.92 – 0.94)	0.858	−0.38(−2.32 – 1.92)	0.704
Accident deaths mean	0.21(0.04 – 0.40)	**0.020**	0.18(0.03 – 0.35)	**0.024**	0.23(0.08 – 0.39)	**0.001**						
Autopsy rate mean	0.09(−0.06 – 0.25)	0.296	0.15(−0.04 – 0.35)	0.131	0.12(−0.10 – 0.33)	0.226	0.14(−0.30 – 0.64)	0.524	0.07(−0.46 – 0.68)	0.805	−0.25(−0.81 – 0.37)	0.392
Ill defined deaths mean	0.01(−0.11 – 0.13)	0.877	−0.03(−0.10 – 0.07)	0.554	−0.02(−0.11 – 0.08)	0.469	0.08(−0.22 – 0.40)	0.587	0.21(−0.09 – 0.55)	0.188	0.10(−0.06 – 0.33)	0.342
Suicide deaths mean							1.28(0.36 – 2.27)	**0.013**	1.31(0.33 – 2.32)	**0.016**	2.14(0.82 – 3.40)	** < 0·001**
R^2^ Nagelkerke	0.653		0.661		0.831		0.433		0.585		0.746	
AIC	104.107		122.932		126.306		140.558		161.551		172.026	

Bold denotes significance at p = 0.05

**Table 10:** Gee model: standard model with EU only

	Suicide deaths			Accident deaths		
*Predictors*	*Estimates*	*CI*	*p*	*Estimates*	*CI*	*p*
(Intercept)	4.90	0.47 – 9.33	**0.030**	8.05	−0.94 – 17.04	0.079
Time	−0.19	−0.53 – 0.15	0.276	−0.44	−1.07 – 0.20	0.180
Ill defined deaths	−0.06	−0.09 – −0.02	**0.001**	0.22	0.10 – 0.34	** < 0·001**
Accident deaths	0.20	0.05 – 0.34	**0.008**			
Autopsy rate	0.11	−0.03 – 0.25	0.121	0.11	−0.17 – 0.39	0.455
Drug use disorder deaths	0.40	0.21 – 0.58	** < 0·001**	−0.24	−0.83 – 0.36	0.431
Suicide deaths				1.18	0.38 – 1.98	**0.004**
N	23 _Countrynew_			23 _Countrynew_		
Observations	110			110		

Bold denotes significance at p = 0.05

**Table 11:** Gee model, split according to alcohol and illicit drugs

*Predictors*	Accident deaths			Suicide deaths			Suicide deaths			Accident deaths		
	*Estimates*	*CI*	*p*	*Estimates*	*CI*	*p*	*Estimates*	*CI*	*p*	*Estimates*	*CI*	*p*
(Intercept)	16.50	12.58 – 21.65	** < 0·001**	7.05	5.39 – 9.21	** < 0·001**	7.52	4.21 – 10.82	** < 0·001**	17.14	7.79 – 26.48	** < 0·001**
Time	0.96	0.93 – 0.99	**0.003**	0.97	0.93 – 1.00	**0.042**	−0.52	−0.86 – −0.19	**0.002**	−1.59	−2.24 – −0.94	** < 0·001**
Ill defined deaths	1.00	1.00 – 1.01	**0.001**	0.99	0.99 – 1.00	**0.009**	−0.06	−0.09 – −0.03	** < 0·001**	0.13	0.03 – 0.22	**0.007**
Suicide deaths	1.05	1.04 – 1.06	** < 0·001**							0.99	0.38 – 1.60	**0.001**
Autopsy rate	1.00	1.00 – 1.01	0.256	1.00	0.99 – 1.02	0.606	−0.04	−0.05 – −0.02	** < 0·001**	0.10	−0.00 – 0.20	0.051
Illicit drug deaths	0.98	0.94 – 1.02	0.302	1.05	1.02 – 1.08	**0.002**						
Accident deaths				1.02	1.01 – 1.03	** < 0·001**	0.20	0.11 – 0.29	** < 0·001**			
Alcohol deaths							1.06	0.62 – 1.50	** < 0·001**	0.85	−0.25 – 1.95	0.132
N	38 _Countrynew_			38 _Countrynew_			38 _Countrynew_			38 _Countrynew_		
Observations	207			207			208			208		

Bold denotes significance at p = 0.05

**Table 12:** Model, split ill-definied diseases and injuries

*Predictors*	Accident deaths			Suicide deaths			Accident deaths			suicide deaths		
	*Estimates*	*CI*	*p*	*Estimates*	*CI*	*p*	*Estimates*	*CI*	*p*	*Estimates*	*CI*	*p*
(Intercept)	18.13	9.91 – 26.35	** < 0·001**	5.33	4.57 – 6.23	** < 0·001**	14.48	4.54 – 24.41	**0.004**	5.90	2.80 – 8.99	** < 0·001**
Time	−1.49	−2.12 – −0.86	** < 0·001**				−1.28	−2.05 – −0.52	**0.001**	−0.50	−0.82 – −0.18	**0.002**
Ill-defined injuries	0.83	0.08 – 1.59	**0.030**	0.96	0.93 – 0.99	**0.004**						
Suicide deaths	1.26	0.70 – 1.82	** < 0·001**				1.37	0.86 – 1.88	** < 0·001**			
Autopsy rate	0.04	−0.07 – 0.15	0.516	1.01	1.00 – 1.02	0.104	0.12	0.00 – 0.24	**0.046**	−0.04	−0.06 – −0.02	** < 0·001**
Drug use disorder deaths	−0.18	−0.54 – 0.19	0.348	1.03	1.02 – 1.05	** < 0·001**	−0.11	−0.56 – 0.34	0.638	0.60	0.40 – 0.80	** < 0·001**
Accident deaths				1.02	1.01 – 1.03	** < 0·001**				0.25	0.17 – 0.34	** < 0·001**
Ill-defined diseases							0.13	0.01 – 0.25	**0.029**	−0.05	−0.08 – −0.01	**0.007**
N	38 _Countrynew_			38 _Countrynew_			38 _Countrynew_			38 _Countrynew_		
Observations	207			207			209			209		

Bold denotes significance at p = 0.05

**Table 13:** Model, anti depressant use

*Predictors*	Accident deaths			Suicide deaths		
	*Estimates*	*CI*	*p*	*Estimates*	*CI*	*p*
(Intercept)	17.85	7.65 – 28.05	**0.001**	2.25	−3.13 – 7.63	0.413
Time	−0.63	−1.65 – 0.39	0.225	−0.23	−0.73 – 0.27	0.367
Autopsy rate	0.14	−0.13 – 0.42	0.307	0.17	0.02 – 0.31	**0.023**
Anti depressants	−0.08	−0.14 – −0.03	**0.004**	0.04	0.01 – 0.07	**0.015**
Suicide deaths	0.88	0.09 – 1.67	**0.028**			
Accident deaths				0.26	0.06 – 0.45	**0.010**
N	17 _Countrynew_			17 _Countrynew_		
Observations	85			85		

Bold denotes significance at p = 0.05

**Table 14:** Gee: WHO high quality only

	Suicide deaths			Accident deaths		
*Predictors*	*Estimates*	*CI*	*p*	*Estimates*	*CI*	*p*
(Intercept)	6.05	2.66 – 9.45	** < 0·001**	10.04	0.57 – 19.51	**0.038**
Time	−0.46	−0.84 – −0.08	**0.018**	−0.91	−1.60 – −0.22	**0.009**
Ill defined deaths	−0.07	−0.10 – −0.03	** < 0·001**	0.21	0.09 – 0.33	**0.001**
Accident deaths	0.27	0.19 – 0.35	** < 0·001**			
Autopsy rate	−0.03	−0.06 – −0.00	**0.031**	0.08	−0.03 – 0.19	0.135
Drug use disorder deaths	0.49	0.29 – 0.70	** < 0·001**	−0.21	−0.64 – 0.23	0.349
Suicide deaths				1.53	1.01 – 2.04	** < 0·001**
N	30 _Countrynew_			30 _Countrynew_		
Observations	178			178		

Bold denotes significance at p = 0.05

**Table 15:** GLM WHO high quality only

*Predictors*	Suicide: 2022–2012		Suicide: 2011–2002		Suicide: 2001–1992		Accidents:2022–2012		Accidents:2011–2002		Accidents: 2001–1992	
	*Estimates*	*p*	*Estimates*	*p*	*Estimates*	*p*	*Estimates*	*p*	*Estimates*	*p*	*Estimates*	*p*
Drug use disorder deaths mean	0.45(0.17 – 0.76)	**0.001**	0.36(0.02 – 0.78)	**0.031**	0.62(0.11 – 1.18)	**0.015**	−0.23(−0.89 – 0.55)	0.531	−0.08(−0.92 – 0.95)	0.877	−0.08(−1.93 – 1.87)	0.928
Accident deaths mean	0.25(0.12 – 0.40)	** < 0·001**	0.25(0.15 – 0.37)	** < 0·001**	0.24(0.12 – 0.38)	** < 0·001**						
Autopsy rate mean	−0.07(−0.10 – −0.03)	** < 0·001**	−0.05(−0.10 – 0.03)	0.154	0.14(−0.05 – 0.33)	0.129	0.08(−0.07 – 0.32)	0.387	−0.03(−0.35 – 0.47)	0.870	−0.21(−0.72 – 0.37)	0.423
Ill defined deaths mean	−0.02(−0.09 – 0.06)	0.641	−0.07(−0.13 – −0.00)	0.057	−0.06(−0.11 – 0.01)	**0.023**	0.22(−0.05 – 0.53)	0.122	0.28(−0.02 – 0.59)	0.056	0.18(0.04 – 0.37)	**0.038**
Suicide deaths mean							1.42(0.45 – 2.45)	**0.006**	1.75(0.76 – 2.77)	** < 0·001**	2.02(0.90 – 3.13)	** < 0·001**
R^2^ Nagelkerke	0.669		0.687		0.824		0.488		0.638		0.765	
AIC	143.635		156.429		147.359		200.831		212.104		202.202	

Bold denotes significance at p = 0.05

**Table 16:** GLM Bivariate models suicide | accidents ~ autopsy rate

Predictors	Suicide: 2022–2012		Suicide: 2011–2002		Suicide: 2001–1992		Suicide: 1993–1982		Accidents: 2022–2012		Accidents: 2011–2002		Accidents: 2001–1992		Accidents: 1993–1982	
	Estimates	p	Estimates	p	Estimates	p	Estimates	p	Estimates	p	Estimates	p	Estimates	p	Estimates	p
Autopsy rate	0.08(−0.05 – 0.25)	0.124	0.35(0.17 – 0.56)	** < 0.001**	0.55(0.31 – 0.82)	** < 0.001**	0.49(0.37 – 0.62)	** < 0.001**	0.54(0.21 – 0.98)	**0.002**	1.09(0.55 – 1.76)	** < 0.001**	0.98(0.41 – 1.66)	**0.005**	0.78(0.34 – 1.23)	**0.001**
Observations	34		36		38		33		34		36		38		33	
R^2^ Nagelkerke	0.032		0.299		0.385		0.593		0.355		0.439		0.295		0.264	
AIC	196.342		227.227		261.738		205.260		245.643		293.089		328.707		281.611	

Bold denotes significance at p = 0.05

**Table 17:** GLM bivariate models suicide | accidents ~ ill defined deaths

Predictors	Suicide: 2022–2012		Suicide: 2011–2002		Suicide: 2001–1992		Suicide: 1993–1979		Accidents: 2022–2012		Accidents: 2011–2002		Accidents: 2001–1992		Accidents: 1993–1979	
	Estimates	*p*	Estimates	*p*	Estimates	*p*	Estimates	*p*	Estimates	*p*	Estimates	*p*	Estimates	*p*	Estimates	*p*
Ill defined deaths	−0.03(−0.05 – −0.00)	**0.004**	−0.03(−0.08 – 0.04)	0.135	0.04(−0.04 – 0.14)	0.356	−0.09(−0.14 – −0.02)	** < 0.001**	0.05(−0.03 – 0.18)	0.315	0.11(−0.08 – 0.36)	0.226	0.33(0.13 – 0.56)	**0.003**	−0.06(−0.25 – 0.21)	0.546
Observations	47		49		48		46		47		49		48		46	
R^2^ Nagelkerke	0.081		0.025		0.025		0.095		0.037		0.035		0.257		0.006	
AIC	262.368		312.354		341.106		320.176		327.005		388.595		408.288		394.935	

Bold denotes significance at p = 0.05

**Table 18:** GLM bivariate models suicide | accidents ~ drug use disorder deaths

Predictors	Suicide: 2022–2012		Suicide: 2011–2002		Suicide: 2001–1992		Suicide: 1993–1979		Accidents: 2022–2012		Accidents: 2011–2002		Accidents: 2001–1992		Accidents: 1993–1979	
	Estimates	*p*	Estimates	*p*	Estimates	*p*	Estimates	*p*	Estimates	*p*	Estimates	*p*	Estimates	p	Estimates	*p*
Drug use disorder deaths	0.90(0.62 – 1.20)	** < 0.001**	1.29(0.66 – 2.04)	** < 0.001**	2.04(1.25 – 2.96)	** < 0.001**	3.49(1.32 – 6.12)	** < 0.001**	0.76(0.20 – 1.41)	**0.015**	1.40(0.38 – 2.70)	**0.013**	2.57(1.45 – 3.88)	** < 0.001**	1.11(−2.22 – 5.08)	0.451
R^2^ Nagelkerke	0.542		0.392		0.582		0.288		0.170		0.240		0.429		0.013	

Bold denotes significance at p = 0.05

**Table 19:** GLM bivariate models suicide | accidents ~ accidents|suicide

Predictors	Suicide: 2022–2012		Suicide: 2011–2002		Suicide: 2001–1992		Suicide: 1993–1979		Accidents: 2022–2012		Accidents: 2011–2002		Accidents: 2001–1992		Accidents: 1993–1979	
	Estimates	p	Estimates	p	Estimates	p	Estimates	p	Estimates	p	Estimates	p	Estimates	p	Estimates	p
Accident deaths	0.20(0.09 – 0.33)	**0.001**	0.24(0.14 – 0.37)	** < 0.001**	0.32(0.21 – 0.46)	** < 0.001**	0.26(0.13 – 0.39)	** < 0.001**								
Suicide deaths									1.13(0.56 – 1.72)	**0.001**	1.60(1.14 – 2.12)	** < 0.001**	1.46(1.06 – 1.89)	** < 0.001**	0.89(0.27 – 1.53)	**0.013**
R^2^ Nagelkerke	0.228		0.397		0.467		0.238		0.306		0.574		0.600		0.191	

Bold denotes significance at p = 0.05
